# Retraction: Andrographolide Suppress Tumor Growth by Inhibiting TLR4/NF-κB Signaling Activation in Insulinoma

**DOI:** 10.7150/ijbs.66938

**Published:** 2021-10-08

**Authors:** International Journal of Biological Sciences

This article (Andrographolide Suppress Tumor Growth by Inhibiting TLR4/NF-κB Signaling Activation in Insulinoma. Qian-Qian Zhang, Yi Ding, Yan Lei, et al. Int J Biol Sci 2014; 10(4): 404-414) has been retracted due to concerns on multiple image duplications as identified by PubPeer users. This raises concerns about the integrity of the data presented in the paper.

